# Supplementary material for: CCR1 mediates Müller cell activation and photoreceptor cell death in macular and retinal degeneration
Source: eLife. 2023 Oct 30;12:e81208. doi: 10.7554/eLife.81208 (PMC10615370; doi:10.7554/eLife.81208)
Supplement: MDAR checklist [file elife-81208-mdarchecklist1.docx]

**Materials Design Analysis Reporting (MDAR)**

**Checklist for Authors**

The [MDAR framework](https://osf.io/xfpn4/) establishes a minimum set of requirements in transparent reporting mainly applicable to studies in the life sciences.

*eLife* asks authors to **provide detailed information within their article** to facilitate the interpretation and replication of their work. Authors can also upload supporting materials to comply with relevant reporting guidelines for health-related research (see [EQUATOR Network](http://www.equator-network.org/%20)), life science research (see the [BioSharing Information Resource](http://biosharing.org/)), or animal research (see the [ARRIVE Guidelines](http://www.plosbiology.org/article/info:doi/10.1371/journal.pbio.1000412) and the [STRANGE Framework](https://doi.org/10.1038/d41586-020-01751-5); for details, see *eLife*’s [Journal Policies](https://reviewer.elifesciences.org/author-guide/journal-policies)). Where applicable, authors should refer to any relevant reporting standards materials in this form.

For all that apply, please note **where in the article** the information is provided. Please note that we also collect information about data availability and ethics in the submission form.

**Materials:**

| **Newly created materials** | **Indicate where provided: section/figure legend** | **N/A** |
| --- | --- | --- |
| The manuscript includes a dedicated "materials availability statement" providing transparent disclosure about availability of newly created materials including details on how materials can be accessed and describing any restrictions on access. |  | N/A |
|  |  |  |
| **Antibodies** | **Indicate where provided: section/figure legend** | **N/A** |
| For commercial reagents, pro  vide supplier name, catalogue number and [RRID](https://scicrunch.org/resources), if available.  mouse anti-rhodopsin (ab3267)  mouse anti-RPE65 (ab13826)  rabbit anti-4 hydroxynonenal (HNE) (ab46545)  rat anti-cd11b antibody (ab64347)  mouse anti-human CCR1 (mab145)  rat anti-mouse CCR1 (mab5986)  rat anti-mouse-CCR5 (ab11466)  rabbit anti-mouse CCR2 (NBP2-67700)  rabbit anti-mouse GFAP (ab64347)  rabbit anti-mouse Iba1 (ab153696).  donkey anti-mouse IgG-Alexa Fluor 488 (ab150109) donkey anti-rat IgG-Alexa Fluor 555 (ab150154)  goat anti-rabbit IgG-Alexa Fluor 488 (ab150085)  anti-human CCR1 antibody (mab145) | Material and Methods section  1. Macrophage co-cultures with mouse retinal explants - Lines 569-573  2. Immunohistochemistry-  Lines 669-677 |  |
|  |  |  |
| **DNA and RNA sequences** | **Indicate where provided: section/figure legend** | **N/A** |
| Short novel DNA or RNA including primers, probes: Sequences should be included or deposited in a public repository. | Material and Methods section  Reverse transcription and real-time quantitative PCR (qPCR) -  Lines :590 |  |

| Gene | Forward (5’-3’) | Reverse (5’-3’) |  |
| --- | --- | --- | --- |
| human *GAPDH*  human CXCL10  human CCL17  human CD163 | AACAGCCTCAAGATCATCAGC  GGTGAGAAGAGATGTCTGAATCC  AGGGATGCCATCGTTTTTGTAA  CAGTGCAGAAAACCCCACAA | GGATGATGTTCTGGAGAGCC  GTCCATCCTTGGAAGCACTGCA  GCTTCAAGACCTCTCAAGGCT  AAAGGATGACTGACGGGATGA | |
| human *MCP4* | ATCTCCTTGCAGAGGCTGAA | CTTCTCCTTTGGGTCAGCAC | |
| human *MPIF1* | TTTGAAACGAACAGCGAGTG | CAGCATTCTCACGCAAACC | |
| human *HCC1* | ATACAGCTAAAGTTGGTGGGGG | TGGTGATGAAGACAATTCCGGG | |
| human *CCR1* | AAGTCCCTTGGAACCAGAGAGAAG | CCAACCAGGCCAATGACAAA | |
| mouse *Gapdh* | AACTTTGGCATTGTGGAAGG | ACACATTGGGGGTAGGAACA | |
| mouse *Ccr1* | GTTGGGACCTTGAACCTTGA | CCCAAAGGCTCTTACAGCAG | |
| mouse *Ccr2* | GAAGAGGGCATTGGATTCAC | TATGCCGTGGATGAACTGAG | |
| mouse *Ccr5* | TCTCCTAGCCAGAGGAGGTG | TGTCATAGCTATAGGTCGGAACTG | |
| mouse *Adgre1** | GCATCATGGCATACCTGTTC | AGTCTGGGAATGGGAGCTAA | |
| mouse *Ccl2* | AGGTCCCTGTCATGCTTCTG | TCTGGACCCATTCCTTCTTG | |
| mouse *Cxcl1* | GACCATGGCTGGGATTCACC | CCAAGGGAGCTTCAGGGTCA | |
| mouse *Cxcl10* | CATCCCTGCGAGCCTATCC | CATCTCTGCTCATCATTCTTTTTCA | |

|  |  |  |
| --- | --- | --- |
| **Cell materials** | **Indicate where provided: section/figure legend** | **N/A** |
| Cell lines: Provide species information, strain. Provide accession number in repository OR supplier name, catalog number, clone number, OR RRID. |  | N/A |
| Primary cultures: Provide species, strain, sex of origin, genetic modification status.  Human monocyte/ macrophage cells, from male or female origin | Material and Methods section  Preparation of monocytes and macrophages-Lines:518 |  |
|  |  |  |
| **Experimental animals** | **Indicate where provided: section/figure legend** | **N/A** |
| Laboratory animals or Model organisms: Provide species, strain, sex, age, genetic modification status. Provide accession number in repository OR supplier name, catalog number, clone number, OR RRID.  Albino BALB/c mice, 6-week-old, male or female,(BALB/C OLAHSD)  C57BL/6, 6-week-old, male or female,(C57BL/6JOLAHSD) | Material and Methods section  Macrophage co-cultures with mouse retinal explants- Line 556  Photo-oxidative retinal injury and intravitreal injections-  Line 595 |  |
| Animal observed in or captured from the field: Provide species, sex, and age where possible. |  | N/A |
|  |  |  |
| **Plants and microbes** | **Indicate where provided: section/figure legend** | **N/A** |
| Plants: provide species and strain, ecotype and cultivar where relevant, unique accession number if available, and source (including location for collected wild specimens). |  | N/A |
| Microbes: provide species and strain, unique accession number if available, and source. |  | N/A |
|  |  |  |
| **Human research participants** | **Indicate where provided: section/figure legend) or state if these demographics were not collected** | **N/A** |
| If collected and within the bounds of privacy constraints report on age, sex, gender and ethnicity for all study participants.  A total of 33 patients with AMD (14 women and 19 men) 77.1 ± 3 years of age (range: 63-95 years) were recruited.  Ethnicity: White | Material and Methods section  Patients-line 505 |  |

**Design:**

| **Study protocol** | **Indicate where provided: section/figure legend** | **N/A** |
| --- | --- | --- |
| If the study protocol has been pre-registered, provide DOI. For clinical trials, provide the trial registration number OR cite DOI. |  | N/A |
|  |  |  |
| **Laboratory protocol** | **Indicate where provided: section/figure legend** | **N/A** |
| Provide DOI OR other citation details if detailed step-by-step protocols are available. |  | N/A |
|  |  |  |
| **Experimental study design (statistics details) *** | | |
| **For in vivo studies: State whether and how the following have been done** | **Indicate where provided: section/figure legend. If it could have been done, but was not, write “not done”** | **N/A** |
| Sample size determination | Sample size was determined based on similarly researches, previously done in our laboratory in which sample size has been determined statistically before experiment. |  |
| Randomisation |  | **N/A** |
| Blinding |  | N/A |
| Inclusion/exclusion criteria |  | N/A |
|  |  |  |
| **Sample definition and in-laboratory replication** | **Indicate where provided: section/figure legend** | **N/A** |
| State number of times the experiment was replicated in the laboratory.  Monocyte injection: n=7  Validation of Macrophage polarization: n=5  Macrophages injection: n=8  Macrophages in-vitro effect: n=6  Macrophages in-vitro characterization: n=5  CCR1 ligands expression in M2a: n=6  CCR1/CCR2/CCR5 is upregulated in a mouse model of photic injury: n=6  Ccr1 is upregulated in both rd10 mice and senescent mice: n=7-8  In-vivo inhibition of CCR1: n=10  Real-time qPCR to evaluate retinal inflammation: n=5-6  Effect of CCR1 inhibition in M2a macrophages: n=3-5 | Supplementary Figure S1: Line 1125  Figure 1: Line 1135  Figure 1: Line 1141  Figure 2: Line 1189  Figure 3: Line 1205  Supplementary Figure S3: Line 1223  Figure 4: Line 1238  Supplementary Figure S4: Line 1247  Figure 5: Lines 1270-1275  Figure 6: Lines 1286  Figure 6: Lines 1292, 1297,1299  Figure 7: Lines 1308,1310,1313 |  |
| Define whether data describe technical or biological replicates.  Biological replicates |  |  |
|  |  |  |
| **Ethics** | **Indicate where provided: section/submission form** | **N/A** |
| Studies involving human participants: State details of authority granting ethics approval (IRB or equivalent committee(s), provide reference number for approval.  Ethical approval for all experimental protocols and studies involving human subjects were provided by the Local Committee on Research Involving Human Subjects of the Hebrew University-Hadassah Medical School, the Helsinki Committee of Hadassah Medical Organization, and the Israel Ministry of Health's Helsinki Committee for Genetic Experiments on Human Subjects (File # 22-03.08.07). | Ethics approval and consent to participate section |  |
| Studies involving experimental animals: State details of authority granting ethics approval (IRB or equivalent committee(s), provide reference number for approval.  Ethical approval for all protocols involving animals was approved by the Authority for Biological and Biomedical Models (ABBM) and the University Ethics Committee for the Care and Use of Laboratory Animals at the Hebrew University, which is certified by the Association for Assessment and Accreditation of Laboratory Animal Care (AAALAC) (ethical approval number: MD-16-14796-3, NIH approval number: OPRR-A01-5011). | Ethics approval and consent to participate section |  |
| Studies involving specimen and field samples: State if relevant permits obtained, provide details of authority approving study; if none were required, explain why. |  | N/A |
|  |  |  |
| **Dual Use Research of Concern (DURC)** | **Indicate where provided: section/submission form** | **N/A** |
| If study is subject to dual use research of concern regulations, state the authority granting approval and reference number for the regulatory approval. |  | N/A |

**Analysis:**

| **Attrition** | **Indicate where provided: section/figure legend** | **N/A** |
| --- | --- | --- |
| Describe whether exclusion criteria were pre-established. Report if sample or data points were omitted from analysis. If yes, report if this was due to attrition or intentional exclusion and provide justification.  Values over 2 standard deviations from the average were excluded from statistical analysis. |  |  |
|  |  |  |
| **Statistics** | **Indicate where provided: section/figure legend** | **N/A** |
| Describe statistical tests used and justify choice of tests.  The appropriate statistical tests were used based on the results of a test for normalcy and the sample distribution and parameters. The biostatistical software package InStat (GraphPad Software, San Diego, CA) was used for data analysis. Data were analyzed using a one-way ANOVA followed by the Tukey-Kramer post-hoc test or an unpaired Student’s t-test, where appropriate. Details of the number of replicates and the specific statistical test used are provided in the individual figure legends. Values over 2 standard deviations from the average were excluded from statistical analysis. The results are presented as the mean fold change ± the standard error of the mean (SEM). | Statistical analysis section |  |
|  |  |  |
| **Data availability** | **Indicate where provided: section/submission form** | **N/A** |
| For newly created and reused datasets, the manuscript includes a data availability statement that provides details for access (or notes restrictions on access). |  | N/A |
| When newly created datasets are publicly available, provide accession number in repository OR DOI and licensing details where available. |  | N/A |
| If reused data is publicly available provide accession number in repository OR DOI, OR URL, OR citation. |  | N/A |
|  |  |  |
| **Code availability** | **Indicate where provided: section/figure legend** | **N/A** |
| For any computer code/software/mathematical algorithms essential for replicating the main findings of the study, whether newly generated or re-used, the manuscript includes a data availability statement that provides details for access or notes restrictions. |  | N/A |
| Where newly generated code is publicly available, provide accession number in repository, OR DOI OR URL and licensing details where available. State any restrictions on code availability or accessibility. |  | N/A |
| If reused code is publicly available provide accession number in repository OR DOI OR URL, OR citation. |  | N/A |

**Reporting:**

The MDAR framework recommends adoption of discipline-specific guidelines, established and endorsed through community initiatives.

| **Adherence to community standards** | **Indicate where provided: section/figure legend** | **N/A** |
| --- | --- | --- |
| State if relevant guidelines (e.g., ICMJE, MIBBI, ARRIVE, STRANGE) have been followed, and whether a checklist (e.g., CONSORT, PRISMA, ARRIVE) is provided with the manuscript. |  | N/A |

* We provide the following guidance regarding transparent reporting and statistics; we also refer authors to [Ten common statistical mistakes to watch out for when writing or reviewing a manuscript](https://doi.org/10.7554/eLife.48175).

**Sample-size estimation**

- You should state whether an appropriate sample size was computed when the study was being designed
- You should state the statistical method of sample size computation and any required assumptions
- If no explicit power analysis was used, you should describe how you decided what sample (replicate) size (number) to use

**Replicates**

- You should report how often each experiment was performed
- You should include a definition of biological versus technical replication
- The data obtained should be provided and sufficient information should be provided to indicate the number of independent biological and/or technical replicates
- If you encountered any outliers, you should describe how these were handled
- Criteria for exclusion/inclusion of data should be clearly stated
- High-throughput sequence data should be uploaded before submission, with a private link for reviewers provided (these are available from both GEO and ArrayExpress)

**Statistical reporting**

- Statistical analysis methods should be described and justified
- Raw data should be presented in figures whenever informative to do so (typically when N per group is less than 10)
- For each experiment, you should identify the statistical tests used, exact values of N, definitions of center, methods of multiple test correction, and dispersion and precision measures (e.g., mean, median, SD, SEM, confidence intervals; and, for the major substantive results, a measure of effect size (e.g., Pearson's r, Cohen's d)
- Report exact p-values wherever possible alongside the summary statistics and 95% confidence intervals. These should be reported for all key questions and not only when the p-value is less than 0.05.

**Group allocation**

- Indicate how samples were allocated into experimental groups (in the case of clinical studies, please specify allocation to treatment method); if randomization was used, please also state if restricted randomization was applied
- Indicate if masking was used during group allocation, data collection and/or data analysis
